# Minimally invasive single-site surgery for the digestive system: A technological review

**DOI:** 10.4103/0972-9941.72381

**Published:** 2011

**Authors:** Parag W Dhumane, Michele Diana, Joel Leroy, Jacques Marescaux

**Affiliations:** IRCAD/EITS, Hôpitaux Universitaires, 1 Place de l’Hôpital, 67091 Strasbourg Cedex, France

**Keywords:** Laparoendoscopic single site surgery, minimally invasive single site surgery, single-incision laparoscopic surgery, single port access, surgical technology

## Abstract

Minimally Invasive Single Site (MISS) surgery is a better terminology to explain the novel concept of scarless surgery, which is increasingly making its way into clinical practice. But, there are some difficulties. We review the existing technologies for MISS surgery with regards to single-port devices, endoscope and camera, instruments, retractors and also the future perspectives for the evolution of MISS surgery. While we need to move ahead cautiously and wait for the development of appropriate technology, we believe that the “Ultimate form of Minimally Invasive Surgery” will be a hybrid form of MISS surgery and Natural Orifice Transluminal Endoscopic Surgery, complimented by technological innovations from the fields of robotics and computer-assisted surgery.

## INTRODUCTION

Laparoscopic surgery is a well-established alternative to open surgery across various disciplines. Although the magnitude of impact varies by procedure, in general, the benefits of laparoscopy on postoperative pain, cosmesis, hospital stay and convalescence are widely recognized.[1]

The benefits of surgical treatment of disease have always been viewed as being obtained with a certain acceptable level of pain and trauma to the patient. Minimizing this untoward effect of any surgical procedure has been a driving force of laparoscopy since its inception in the early 1900s.[2,3] Natural Orifice Transluminal Endoscopic Surgery (NOTES™) and minimally invasive single-site (MISS) surgery are the recently conceptualized forms of minimal access surgery. To correctly explain this concept of scarless surgery, which aims at inflicting minimum possible trauma to the patient regardless of the surgical specialty, we propose this terminology – MISS surgery. This term will be used subsequently in this article. In a comment published in the BJUI, the experts feel that NOTES™ and MISS surgery are complementary techniques, with “scarless” surgery being the final goal. Experts are of the opinion that “pure” NOTES™ should be vigorously promoted as a research interest, whereas MISS surgery should be clinically used in selected patients.[4] NOTES™ looks like a more futuristic and fundamentally paradigm-changing concept than MISS surgery.[5]

Several reports demonstrated that reducing the number and size of the laparoscopic trocars has an effect on the surgical outcome in terms of postoperative pain and cosmetic results.[6,7] There have been many reports of single-incision appendectomy since Begin (1993) described the first single-incision appendectomy in children.[8] Navarra *et al*. are credited with performing the first single-incision cholecystectomy in 1997.[9] The initial goals of the development of MISS surgery were to develop a technique that fits within what we believe to be the important criteria to maintain current surgical standards. Availability to surgeons and patients, an eye to costs, a focus on safety and a commitment to developing a technique that would not be prohibitive from a training standpoint were all primary goals. On the same lines, is the rationale behind “single port rescue” of putting an additional port site to maintain safety in MISS surgery.[10]

While media attention may persuade patients to seek out this procedure for an enhanced cosmetic result, we must ensure a safe result. Although industry will be liked to the development of new procedures and instrumentation, surgeons should be the driving force in the development of necessary technology and not vice versa.[10] In a questionnaire-based study of 750 participants divided into three groups of doctors, nurses and general population, there was clear-cut preference for MISS surgery over NOTES™, laparoscopic and open surgery for appendectomy. This justifies great enthusiasm about this technique among the surgical community.[11]

Safety must be the leading focus of any new procedure. This is why MISS surgery had become more of a philosophy than just a technique. However, as we move forward, we need to constantly assess the safety and efficacy as compared with multiport procedures. The benefits at this point in its development, we suspect, may be limited. Patients’ desire for the cosmetic benefit is important, but the price cannot be increased risk or complication.[10]

The described procedures of MISS surgery include cholecystectomies, appendectomies, upper gastrointestinal surgeries, some hepatopancreatobiliary procedures, adrenalectomies, complete spectrum of colorectal surgeries, bariatric, gynecologic and pediatric surgery procedures. Urologists are very enthusiastic in experimenting with MISS surgery and implementing it into clinical practice. The surgeons from our institute (IRCAD, Strasbourg) have demonstrated feasibility in humans of MISS surgery for advanced surgical procedures like sigmoidectomy.[12] To date, however, cholecystectomy appears to be the most common surgical procedure to which significant efforts have been applied toward the development of technique and equipment.[13]

The Laparoendoscopic Single-Site Surgery Consortium for Assessment and Research (LESSCAR) has been formed to serve as an international multidisciplinary *ad hoc* organization to advance the field of MISS surgery. The primary goal of LESSCAR is to develop the necessary techniques and technology to standardize the clinical outcomes of MISS surgery across various disciplines. LESSCAR tried to end the ambiguity about the nomenclature of this technique by publishing the white paper. After extensive deliberations, LESSCAR proposed the term laparoendoscopic single-site (LESS) surgery.[5] On searching for “LESS surgery” in various internet search engines, we concluded that it will be commercially biased to use the term “LESS” surgery for this novel surgical concept. Also, this terminology limits the application of this concept only to the abdomen.

Like any other new surgical technique, MISS surgery will have to pass through two important developmental phases, first is the clinical feasibility and safety and the other is suitable technology to perform this technique. Recently, there has been a huge interest in development and testing of new instruments and equipments for MISS surgery. In this article, we have reviewed the existing and futuristic technologies suitable for the concept of MISS surgery.

We review the technological aspects of MISS surgery under the following headings:

## SINGLE-PORT DEVICES

In an early report from Japan (2001), Kagaya had devised a “Twin port” (10 mm approximately) – quite similar to the single-port device designs we currently have in the market – which can accommodate an endoscope (5 mm) and a grasper (5 mm).The surgeon demonstrated feasibility by performing laparoscopic cholecystectomy with “Twin port” and an additional 5 mm sub-xiphoid trocar in a series of 40 patients, with only three (out of 40) patients needing an additional fourth instrument.[14]

Central to the performance of MISS surgery is the ability to achieve efficient and effective access to the surgical area of interest via a single port of entry. Anticipating ongoing refinements in single-port technology, LESSCAR recognizes that the use of three standard laparoscopic trocars through a single skin incision (with multiple fascial puncture sites) can provide the independence of movement necessary for MISS surgery.[5] This increased circle of entry permits larger triangulation at the abdominal wall, enabling us to move to straight instruments.[10]

MISS surgery can either be performed using a single-port device or individual trocars in the same or independent fascial incisions. The existing single-port devices can be broadly classified on the basis of two components – access and retracting technology [Table 1].

**Table 1:** Classification of single port devices

On the basis of Access	
Gel	e.g. Gelpoint^®^ (Applied Medical)
Multiple channels	e.g. Xcone^®^ (Karl Storz), Endocone^®^ (Karl Storz) Triport^®^ (Advanced surgical), Quadport^®^ (Advanced surgical), OCTO-port^™^ (Dalim surgnet), Glove for MISS surgery.
Structural Access	e.g. SILS^®^ (Covedien), Airseal^®^ (Surgiquest)
**On the basis of retracting technology**	
Sleeve	e.g. Gelpoint^®^ (Applied Medical), Triport^®^ (Advanced surgical), Quadport^®^ (Advanced surgical), OCTOport^™^ (Dalim surgnet), Glove for MISS.
Soft Structural retraction	e.g. SILS^®^ (Covedien).
Rigid structural retraction	e.g. X- cone^®^ (Karl Storz), Endocone^®^ (Karl Storz), Airseal^®^ (Surgiquest)

**Figure 1:**
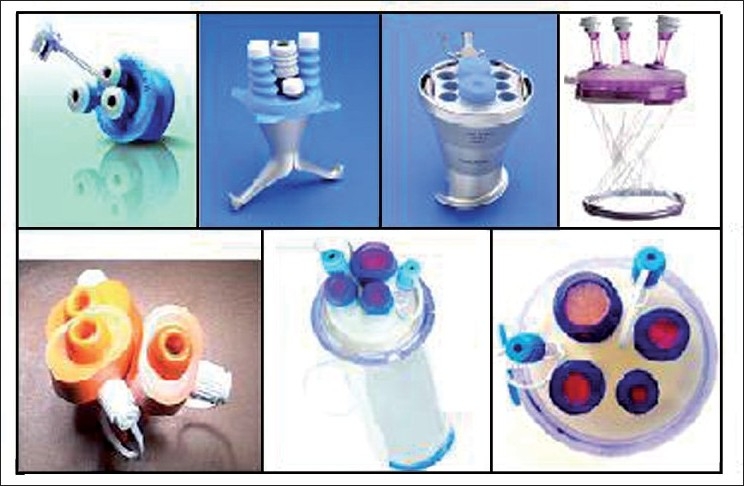
Various single-port devices [from bottom left (clockwise)- Dexide Ports^®^, SILS^™^, (both by Covidien), X-cone^®^, Endocone^®^ (both by Karl Storz), Gelpoint^™^ (Applied Medical), Quadport^®^ and Triport^®^ (both by Advanced Surgical)]

SILS^®^ port (Covidien, Norwalk, CT, USA) is a foam plug that is inserted through a 2-cm fascial incision, which expands once inserted to retract the abdominal wall and prevent & air leakage. Small holes within the foam accommodate 5–12 mm trocars and instruments. Multichannel ports admit three or four instruments. The X-cone^®^ (KARL STORZ GmbH and Co. KG, Tuttlingen, Germany) is a rigid, reusable access port with three operating channels. Cuschieri Endocone^®^ (KARL STORZ GmbH and Co. KG, Tuttlingen, Germany) is a reusable port with multiple channels. GelPOINT^®^ (Applied Medical, Rancho Santa Margarita, CA, USA) a concept initially introduced for hand-assisted laparoscopic surgery, gives a pseudoabdomen platform and has advantages like minimal resistance to external mobility of instrument and maintenance of pneumoperitonium. Triport^®^ and Quadport^®^ (both by Advanced Surgical Concepts, Bray, Ireland; distributed by Olympus) use plastic wound protectors for retraction. AirSeal^®^ (Surgiquest, Orange, CT, USA) is a self-retracting oval cannula with no physical seal, admitting instruments of different shapes and sizes. It maintains pneumoperitonium by creating air vortex with a custom-made insufflator, at times getting noisy.[15,16] There are many studies of a variety of MISS procedures using the surgical glove for maintaining the pneumoperitoneum and port insertion.[17–19] Recently, Dalim Surgnet from South Korea came out with an innovative design for the single-port device called OCTO-port^™^. It utilizes a sleeve-like mechanism for retraction and has a detachable soft silicone cover with a various port mix of choice from one to four channels. It also has a unique feature of smoke filtration during insufflation. It comes in two sizes of 30 mm and 50 mm[20] [Figure 1].

There are various options available in the market for the low-profile trocars apart from the conventional laparoscopic trocars. The Dexide^®^ Ports (Covidien, Norwalk, CT, USA) have been specially designed for MISS surgery. AnchorPort^®^ (Surgiquest, Orange, CT, USA) is a small head trocar with a blunt tip. The Hunt trocars^®^ (Apple Medical Corporation, Marlborough MA, USA) probably have the smallest diameter head available on the market. These are very popular trocars and many surgeons like these trocars because they are inexpensive and have a very low profile. Blunt trocars by Applied Medical (Rancho Santa Margarita, CA, USA) are other options for a low-profile trocar.[16]

Badajoz *et al*. patented an instrument that was 4 cm in diameter and 5 cm long cannula with five soft-sealed orifices on the superior lid to ensure hermetic closure. The central orifice receivesd a 5 mm telescope while the surrounding four 5 mm orifices served to receive different instruments. The authors claimed that the system is particularly appropriate for single-arm robotic surgery in which the telescope and the instruments are carried on just one arm.[21]

## ENDOSCOPE AND CAMERA

### Conventional laparoscope

30° rigid endoscope (5 mm and 10 mm) is the most common endoscope used for MISS surgery today. High-fidelity vision is necessary for intraoperative visualization of anatomic regions and structures. The surgeon should be able to visualize structures from differing perspectives, preferably offline from the axis of the instruments.

### Flexible endoscope

Palanivelu *et al*. described for the first time the feasibility of flexible endoscope for performing transumbilical appendectomy in 10 patients (conversion in four patients).[22] They used an additional 3 mm, rigid, laparoscopic grasper for retraction. Since then, there have been anecdotal reports of use of flexible endoscope for various surgeries like fenestrations of liver cysts,[23] cholecystectomies by saline infiltration gall bladder fossa dissection technique[24] and simple nephrectomy using flexible cystoscope introduced through the 3-cm umbilical incision.[25]

Binenbaum *et al*. share their experience of safe and efficacious use of conventional single- or double-channel flexible endoscope (KARL STORZ GmbH and Co. KGTuttlingen, Germany and Olympus America Inc., Pennsylvania, PA, USA) for MISS cholecystectomy. They used an additional 5 mm trocar, entering by the side of the endoscope through the 15-mm umbilical incision. This technique constitutes a relatively unstable operating platform with obvious lack of triangulation, but had few advantages, like enhanced ergonomics and decreased conflict of instruments with hassle-free vision due to the irrigation systems of flexible endoscopes.[26] We believe that this concept of transumbilical use of flexible endoscope and instrumentation will prove to be a pivotal step in the evolution of MISS surgery.

### Modified laparoscopes

Both Karl Storz endoscopy (Tuttlingen, Germany) and Stryker endoscopy (San Jose, CA, USA) have modified their existing endoscopes to develop 5-mm, 30-degree telescopes that are 50 cm long. The extra length removes the camera head and light cord from the operative field. But, the added length reduces the brightness of the scope and visualization can be poor in larger patients. EndoCAMeleon™ is a 10-mm rigid scope with adjusting knob for selection of viewing direction angle from a range of 0° to 120°. There are operating laparoscopes with 45° or 90°-angled eyepieces with an operating channel (5.5–7 mm) for instruments. They can be of value in MISS surgery to put the endoscope away and increase working space for the instruments.

Another solution for vision in MISS surgery is the EndoEYE™ (Olympus Deutschland GmbH, Hamburg, Germany), a high-definition video laparoscope with a charge-coupled device (CCD) chip at the end of the scope. This scope provides a high-quality image but, it is not compatible with other video platform systems.[16] IDEAL EYES HD™ Articulating Laparoscope (Stryker endoscopy, San Jose, CA, USA) is a 10-mm scope with a facility to toggle between rigid and flexible distal tip with over 100° of flexion in all directions. Eyemax CCD Endoscope™ (Richard Wolf GmbH, Knittlingen, Germany) is a 5–10 mm, 0–30° digital scope with an in-line design, helping to keep the endoscope off the instrument axis.[15]

In a recently published article, the gynecologists concluded that the obvious disadvantage of rigid scopes and their fixed vision were not overcome by the new generation endoscopes and the advantage of adjustable viewing angles. They studied EndoCAMeleon™ (KARL STORZ and Co. KG) and Endoeye™ (EndoEYE LTF-VH, Olympus Deutschland GmbH) for visualization of anterior pelvic structures per vaginally.[27]

### Robotic camera manipulators

To alleviate such problems attributable to a human camera assistant, robotic camera systems such as AESOP^®^ (Computer Motion Inc., Goleta, CA, USA), EndoAssist^®^ (Armstrong Healthcare Ltd., Loudwater, UK) and the Naviot^®^ system (Hitachi Ltd., Tokyo, Japan) have been developed. The Naviot^®^ system is a robotic laparoscope manipulator that consists of a special laparoscope with an optical zoom, a flexible arm, an actuator, a five-bar linkage mechanism and a hand controller with two buttons for zoom and movement of laparoscope in eight directions.[28] But, none of the above systems is free from problems like restricted positioning of the operating table, hindrance in instrument movement, cost, etc.

The researchers demonstrated feasibility and usefulness of a novel and compact robotic camera driver, EndoControl^®^ (Light Endoscope Holder Robot, LER, Grenoble, France) for 5 mm, 30° rigid high-definition laparoscope (EndoEYE™, Olympus Surgical, Orangeburg, NY, USA) introduced through the multichannel port (TriPort, Advanced Surgical Concepts Bray, Ireland). LER affords the surgeon a videoscopic rotational capacity of 360° and a pitch capacity of 70°. It helps to keep the laparoscope steady for prolonged periods without ergonomic challenges to the operator as it occupies less space in the operative field. The study infers that the cumulative benefit of LER during MISS surgery is an improved and unencumbered range of motion with an ability to focus on the operative field wholly and without interference. Control of the LER with foot or voice command obviates communication issues with a bedside assistant and allows the primary surgeon to focus the camera in an optimal, self-directed position. Use of LER overcomes many of the technical obstacles associated with MISS surgery and, with further refinement, might allow more flexibility with the technique.[29]

A compact and lightweight (580 g) laparoscope-manipulating robot by the name of P-arm is developed by the surgeons from Osaka, Japan.[30] This robot is comprised of a manipulator, controlling interface and controller unit. The manipulator is driven by a Stewart–Gough platform equipped with six hydraulic linear actuators with six degrees of freedom. The vertical and transverse movement has a 22° range. The parts of the manipulator are made of materials suitable for medical use and sterilization. The operating table can be tilted without repositioning the manipulator. The laparoscope shaft is attached to the manipulator with small magnets; thus, it can accommodate various types of laparoscopes. The software time delay is just 0.1 s.

### Magnet for camera

In order to rectify the “tunnel vision” due to long endoscope and in-line arrangement of the devices in case of MISS surgery, a laudable attempt has been made by the researchers from the Imperial College, London, to develop a magnet-driven camera powered by LED lights, which looks from above and gives a “stadium view” of the surgical field.[31] This camera can be introduced through a 20-mm incision and the wire tethers of the camera are pulled out of the abdominal cavity using a 2 mm rigid grasper. They concluded that this device gives illumination sufficient to provide reasonable imagery of the nearby abdominal organs. But, the overall image quality was not good. All points on the abdominal wall could be reached via the magnetic control and the camera could be successfully aimed through abdominal palpation. Introduction and retrieval of the device were uncomplicated. Dedicated and specific efforts towards problem solving in the fields of illumination, arrangements of lenses, zoom and focus functions, wireless image transmission, local power source for camera and controllable tilting camera motion and miniaturization, etc. can offer a definitive solution for “vision” in MISS surgery and NOTES™.

Magnets have several advantages over human manual manipulation. They are able to produce a measured force continuously over long periods of time. The force can be directed and exerted through the mucosa and bone without direct contact. Also, they can be made to attract or repel and therefore to push or pull. Fakhry *et al*. demonstrated that the use of the magnetic wireless camera setup with a single external handle is feasible and can provide a wider visual exposure than the classical 30° endoscope.[32]

Movement of the internal Magnetic Anchoring and Guidance System (MAGS) camera is accomplished by manipulation of the external magnet and variable abdominal wall compression.[33] The imaging system is based on a commercially available CCD imager and powered by LED lights. The instruments can be sterilized by a low-temperature method such as hydrogen peroxide gas plasma. The two options for manipulation of an MAGS camera from the exterior are by external magnets or by 14–18-gauge needle anchor systems. It is introduced through a 25-mm incision and wires of the camera exited from one of the ports of the single-port device. The external endoscopic camera needs to be used only for cleaning and magnetic coupling of the MAGS camera. The authors concluded that the use of an MAGS camera results in fewer instrument collisions, improves surgical working space and provides an image comparable to the standard 5 mm laparoscope. Improved ergonomics can be attributed mainly to elimination of the conventional laparoscope from the working space outside the umbilical port. Having a wireless image transfer and local power source for the camera can further benefit this as it will make one more port available for introducing an additional instrument. We feel that the MAGS platform has a potential for changing the paradigms of instrumentation for MISS surgery.

Ultimately, development of wireless, high-resolution CCDs and chip-on-the stick technologies possessing maximal flexibility would represent a true advance.[5]

## INSTRUMENTS

**Figure 2:**
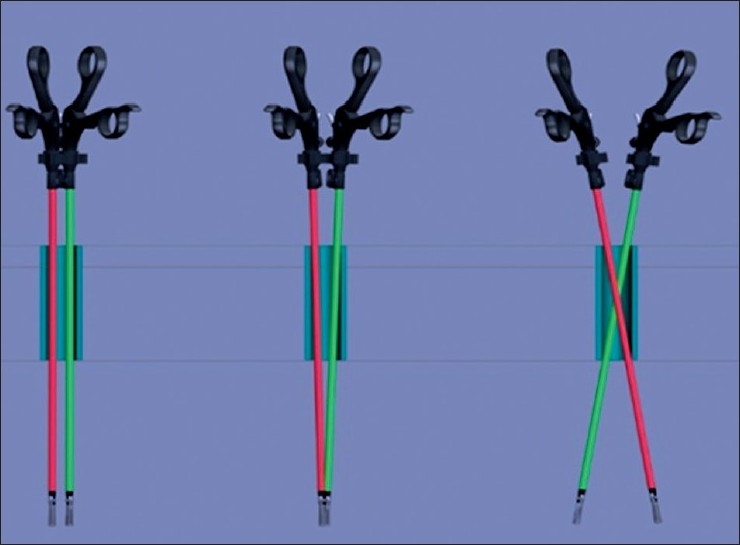
Difficulties with minimally invasive single-site surgery concept (mobility of straight instruments inside rigid bulky trocars depends of the trocars’ diameter)

**Figure 3:**
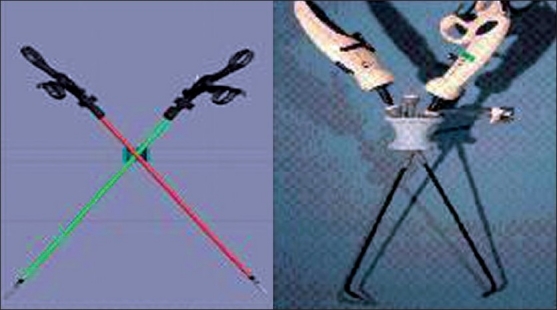
Use of articulated instruments (mobility of straight instruments in short rigid or soft trocars allows to work by crossing handles)

**Figure 4:**
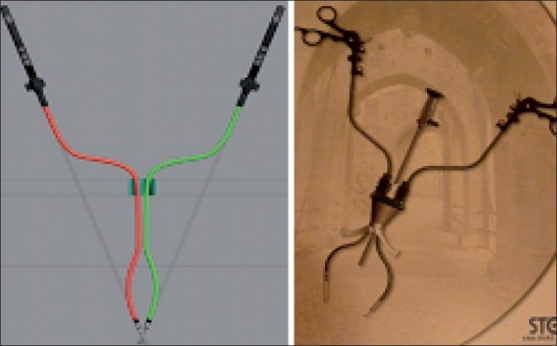
Use of curved instruments (mobility of double-bent instruments in short rigid or soft trocars’ allows to work without crossing handles)

Conflict of instruments, absence of triangulation and difficult retraction are the main technical issues in MISS surgery today. These problems are enhanced if the single-port device is bulky and long as against the compact design [Figure 2]. Many solutions exist to enhance ergonomics and applications of MISS surgery. Use of wristed instruments may improve dexterity, but one may still have to cross the hands while operating. Use of the curved instruments take handles away from each other, at the same time approximating the tips. This phenomenon is explained in Figures 3 and 4.

### Standard (straight) laparoscopic instruments

All conventional laparoscopic instruments can be used for MISS surgery. Using the standard instruments provides reasonable exposure and eliminates the extended time associated with the learning curve at no additional cost. As new techniques develop, their versatility is important.[10]

To increase the surgeon’s confidence, and thereby patient safety, LESSCAR endorses the discretionary adjunctive use of 1.9-mm needle instruments during MISS surgery. By facilitating instrument triangulation, tissue retraction and intracorporeal suturing/knot tying along the lines of standard multiport laparoscopy, these 1.9-mm needle instruments confer three important advantages: enhanced patient safety, increased intraoperative dexterity and an expanded clinical repertoire of MISS surgery with immediate effect. However, the reliability, tensile strength, construction design and variety of needle instruments need improvement.[5]

The Roticulator Endo Dissect™, Roticulator Endo Grasp™ and the Roticulator Endo Mini-Shears™ instruments from Autosuture- Covedien (Norwalk, CT, USA) are of special mention here. These, due to their 80° articulation and 360° rotation of jaws, are of particular help in MISS surgery for retraction and dissection, at the same time helping with conflict and triangulation. For the beginners, we recommend to use these Roticulators™ in combination with a conventional straight laparoscopic instrument. These instruments were developed 20 years ago for laparoscopic procedure and are not well adapted for MISS, although they are useful.

### Curved instruments

The author (JL) has developed a complete range of curved instruments with Karl Storz and Co. KG. The “S-PORTAL^®^ Instruments acc. to Leroy” (Karl Storz and Co.Tuttlingen, Germany) has four curves, which results in the instrument tip and the handle coming in the same line, and this gives the surgeon the feeling of using virtual trocars with triangulation without crossing hands. Apart from this, S-PORTAL^®^ has curved instruments acc. to Cuschieri, Carus and Dapri specifically aimed at solving the problems of triangulation and ergonomics in MISS surgery. Olympus (Orangeburg, NY, USA) offers HiQ™ LS curved 5-mm Hhand instruments. These are reusable rigid instruments with angulated distal and proximal ends and rotator action. Surgiquest™ (Orange, CT, USA) has a range of curved instruments that are designed specifically for MISS surgery.

In an extensively ergonomic-study performed by Manukyan and colleagues, the curved instrument was significantly more efficient with respect to economy of motions and quality of performance in the experimental setup of laparoscopic rectosigmoid resection.[34] The detailed ergonomic investigation was based on four analyzing systems: electromyographic (EMG) measurement, three-dimensional motion analysis with ultrasound tracking system (UTS), simultaneous observation camera and responses on ergonomic questionnaire.

At the end of the experimental study, to compare the ergonomics of conventional, flexible and prebent instruments in MISS surgery, Stolzenberg *et al*. concluded that prebent instruments were less time consuming and had a better maneuverability in comparison to flexible instruments, which was comparable with the performance of conventional straight instruments in MISS surgery.[35]

### Wristed instruments (articulated)

Many articulated instruments are available in the market, which claim to offer added advantages of maneuverability at the tip. Covidien™ (Norwalk, CT, USA) offers SILS™ Hand Instruments specifically designed for MISS surgery in the form of hook, clinch, shears and dissectors. These are strong shaft-wristed instruments with the tip having infinite positions of dynamic articulation with a locking facility and 360° rotation. They are definitely of great help in MISS surgery.

RealHand^®^ High-Dexterity (HD) instruments by Novare Surgical Systems (Cupertino, CA, USA) are designed to mirror the surgeon’s hand direction with the added benefit of tactile feedback. Cambridge Endo^®^ (Framingham, MA, USA) introduced Autonomy™ Laparo-Angle™ instruments – 5 mm roticulators with locking mechanism and articulating tip having 7 degrees of freedom. Encision Inc. (Boulder, CO, USA) brought into market the EnTouch^®^ handles for its AEM^®^ articulating laparoscopic instrument inserts.

Dutta published the first cases of MISS splenectomies and MISS cholecystectomies performed in the pediatric age group using Realhand^®^ HD instruments (Novare Surgical Systems) and a thermal ligation device (Thermaseal^®^; Novare Surgical Systems, Cupertino, CA, USA)).[36]

A significant learning curve was demonstrated when the first-generation novel articulating needle-driver (Cambridge Endo, Framingham, MA, USA) was compared with a conventional laparoscopic needle-driver (Karl Storz and Co. KG) by evaluating the completion of standardized laparoscopic suturing task by novices. This may be secondary due to the complexity of physical maneuvers required for their use.[37]

### “Smart” instruments

The use of multifunction laparoscopic tools can not only help avoid trauma by decreasing removal and reinsertion but can also reduce the number of incisions required to perform a procedure. They also reduce the additional time that is not directly spent in helping the patient in the operating room (OR) and thus reduce the patient risk and costs. A prototype of modular surgical instrument with artificial intelligence was made, which needs further refinement and validation.[38]

There have been reports regarding the laparoscopic use of the Radius^®^ surgical system (Tuebingen Scientific, Tuebingen, Germany), mechanical manipulators (MM) with six degrees of freedom, with ergonomic hand–arm movements and interchangeable endo-effectors.[39,40] Such instruments represent an intermediate stage in the field of minimally invasive surgery between traditional laparoscopic and electromechanic robotical systems. Ishikawa *et al*. report MISS transabdominal preperitoneal hernioplasty (TAPP) using the Radius™ surgical system (Tuebingen Scientific), which allowed the difficult suturing in the abdominal cavity to be accomplished.[41] Using ergonomic handles, the hands and arms of the surgeon are relaxed, avoiding discomfort and fatigue, because all the functions of the instrument are included in the bundle. Obviously, they are not free from shortfalls and need further workup for refinement and cost reduction.

### Instruments for hemostasis

Hemostats for laparoscopic surgery have been in a constant phase of improvisation. We feel that LigaSure™ tissue fusion technology, including LigaSure Advance™ and Valleylab™ mode (CovidienNorwalk, CT, USA), are integral part of the laparoscopic surgeon’s armamentarium and these can become an ideal haemostatic tool for MISS surgery. Sure and rapid means of establishing hemostasis with thermal energy delivery systems, staples, clips and sutures equivalent in efficiency to that of standard multiport laparoscopy are essential. Newer technologies should allow placement of staples, clips and sutures with minimal repetitive instrument exchanges.[5]

Ultrasonic energy is another option for hemostasis and devices like Harmonic™ (Ethicon Endo-Surgery Inc., Cincinnati, OH, USA), AutoSonix™ (Autosuture; CovidienNorwalk, CT, USA) and SonosurgX™ (Olympus, Hamburg, Germany) have been well established in conventional laparoscopy. These instruments are routinely used in MISS surgery.

Ogura and colleagues report the development of a new, compact articulating Ultrasonically Activated Device (USAD) prototype in 5 mm and 10 mm sizes with a bendable tip that offers coagulating dissection performance similar to that of conventional instruments.[42]

### For suturing and anastomosis

Sutures and staplers form the important tools at hand for performing advanced MISS procedures. Salvador Morales-Conde *et al*. demonstrated the successful use of the flexible endostapler (Endo-GIA Roticulator™, Autosuture; Covidien Norwalk, CT, USA) and the Endostitch™ suture system (Autosuture; Covidien Norwalk, CT, USA) introduced through a Quadport™ (Advanced Surgical Concepts) to fashion the entero-colonic anastomosis completely intracorporeally in a case of a transumbilical single-port access right hemicolectomy in a 59-year-old male patient with a neoplasm of the cecum stage IIA (T3, N0).[43]

Danielson *et al*. published the first case report of successful repair of Morgagni’s hernia in a 20-month-old (10 kg) child by MISS surgery using the Endostitch™ suture system (Covidien Norwalk, CT, USA) with extracorporeal knot tying after the initial holding stitch. He used the Triport™ (Advanced Surgical Concepts) and straight-reticulating instrument combination.[44] Use of a 10-mm endoscopic suturing device (SILS™ STITCH, Covidien Norwalk, CT, USA) has been described for MISS Nissen’s Fundoplication.[45]

Endoscopically placed thick internal magnets with external magnet guidance is a feasible and novel approach to creating patent gastroenteral anastomoses without abdominal incisions or sutures.[46] Application of this concept needs further evaluation to make it adaptable for use in clinical MISS surgery.

Jamshidi *et al*. demonstrated the safety and efficacy of magnetic compression anastomosis (magnamosis) device for sutureless, full-thickness intestinal anastomosis with serosal apposition and without leaks in a pig model. They further comment that gradient compression is superior to uniform compression. Mechanical integrity of magnetic anastomosis was similar to, if not better than, staple or suture counterparts. This prompts for clinical trials for its application in humans. Such innovative developments can make the life of MISS surgeons easier.[47]

Novel technologies like compression anostomotic clips, endoluminal alloy rings and electric/laser welding of tissues for anastomosis can add to the armamentarium of anostomotic devices for use in MISS surgery.

## RETRACTORS

The ability to triangulate and grasp tissues firmly enough to allow traction and counter-traction for exposure and dissection is a basic requirement of surgery. One technical challenge of MISS surgery is limited triangulation and retraction of tissues due to confinement of optics and working instruments to a single axis.[5]

Various methods of retraction have been described for MISS cholecystectomy. Navarra *et al*. described the use of transabdominal sutures in hybrid NOTES™ cholecystectomy, which allows continuous extracorporeal manipulation, leaving only a negligible mark.[48] In addition to restricted retraction ability with inability to reposition the retracting suture, another drawback of this method is the potential for intraperitoneal bile leakage and possibly complete tearing of the gallbladder as the needle must pierce the gallbladder wall.

In the study of Schlagger *et al*., the loop of the suture material introduced transabdominally into the peritoneal cavity through a 5-mm trocar was used to retract the gall bladder anteriorly by attaching the endoloop to the fundus.[49] As the gall bladder wall is not pierced, the potential for leakage and complete tearing of the gallbladder is minimized. Nevertheless, this cannot achieve complete superiorly directed retraction. In addition, fastening of the loops may prove challenging in cases of scarred and distended gallbladders.

In Cadeddu’s retraction system using MAGS, two internal magnetic platforms are coupled to either a latex sling or a three-fingered paddle that allows nontraumatic elevation and retraction of organs like the liver and spleen.[50]

Ryou and Thompson describe a modified MAGS retraction system whereby magnet-conjugated clips placed along the inferior edge of the liver are used to accomplish operative retraction.[51] Aside from issues of cost and the necessary learning curve inherent in the introduction of new technology, a significant obstacle to widespread use of MAGS is the exponential decrease in magnetic coupling strength as a function of distance, impairing its use beyond a tissue thickness of 1.5 cm.[49]

Leroy *et al*. (2008) described the use of extracorporeal and intracolonic magnet for retracting the colon and stabilizing the anvil of the circular anastomotic stapler (DST series PCEEA, Autosuture; Covidien Norwalk, CT, USA) while performing single-port sigmoidectomy in the experimental model with survival.[52] They used this trick to perform the first full-laparoscopic MISS sigmoidectomy in human beings.[12]

**Figure 5:**
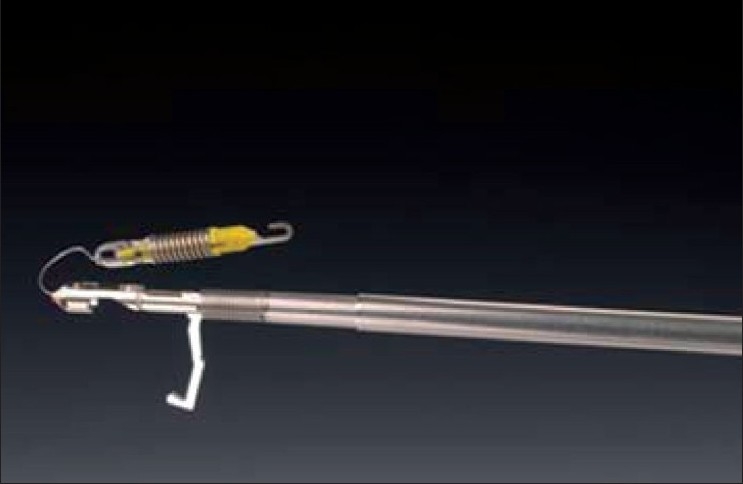
Endograb^®^ retractor (Virtual Ports, Misgav, Israel)

The Endograb™ (Virtual Ports, Misgav, Israel) is an internally anchored retracting device that can be introduced into the abdomen at the outset of the operation through a 5-mm port [Figure 5]. The main advantage of this device is that it not only leaves no visible marks but it can also be anchored superiorly just below the diaphragm, thereby allowing retraction equivalent to that achieved with a designated retracting instrument. This device can be adjusted repeatedly throughout the operation to allow for optimal retraction and is removed at the end of surgery. The Endograb™ retractors have been shown to achieve superior retraction compared with endoloops, with no significant injury to the peritoneal wall.[49]

The researchers from Texas, USA demonstrated the feasibility of MAGS for instruments, retractors and camera during minimal access surgery by performing nephrectomies on two animal models using MAGS and just two trocars. This may expand intracorporeal instrument manipulation substantially beyond current-day capability.[53]

Dominguez *et al*. developed a novel way to use magnets for retraction and enough triangulation for a safe surgery without the need for additional incisions, using magnetic forceps and graspers under the IMANLAP project (“iman” means magnet in Spanish).[54] The possibility of retraction and triangulation from different angles, as if there were multiple access ports with opposite directions, is the principal achievement of this new technique. In addition, the magnetic forceps has unrestricted mobility from the upper abdomen to the pelvis with no need for a new incision. Specially designed Thomas-forceps made with austenitic surgical steel, which has no effect of the magnet field, is used to position and reposition the grasper of the TD-magnet. The study demonstrates successful use of the TD-magnet system for 40 cases of MISS cholecystectomies. They use an 11-mm laparoscope, 0° direction of view, 45° ocular angle, with a 6-mm working channel. Through the working channel, a set of instruments of 5 mm in diameter and longer than 42 cm are passed. The authors claim to have used this system for other MISS surgeries like groin hernia repair, appendicitis and gynecologic procedures.

Uematsu *et al*. published a series of seven patients of MISS colorectal surgeries using a short vascular forceps, 6.0 cm × 2.5 cm, with short handle introduced inside the peritoneal cavity through a 3 cm incision. The forceps are used to catch the intraabdominal structures and are manipulated using a strong columnar magnet from the exterior.[55]

Torre *et al*. describe the technique of liver retraction with 0 silk suture on the right crus above and away from the traversing of the esophagus in MISS-adjustable gastric band placement for obesity. The two threads are exteriorized at two different points using a suture passer.[56]

Hamzaoglu and colleagues describe the “Istanbul Technique” for liver retraction in MISS Nissen’s Fundoplication.[45] The main principle of the technique is an atraumatic suspension of the liver with a hammock-like mechanism using a Penrose drain 8 cm long and 1 cm wide. Two silk sutures in 10 cm lengths were tied to the two different ends of the Penrose drain. These stitches are then brought to the exterior and controlled by clamps.

## FUTURE PERSPECTIVE

Historically, technological advances in instrument design have fueled newer clinical applications, the success of which in turn has necessitated the development of yet newer technology. This self-perpetuating cycle now stands at the threshold of a quantum leap forward due to the most recent developments in NOTES™ and — MISS surgery.

It is estimated that the volume of procedures applicable to MISS surgery may increase by approximately 50% during the next 5 years, with even higher projections of up to 80% in some specialties, with excessive costs potentially limiting applications in specific procedures despite the clinical benefits they might produce. This would hinder progress.[5]

### Instrumentation

The MISS surgery approach has the potential to develop the field of percutaneous intra- and transluminal surgery. By direct percutaneous entry to hollow organs such as the urinary bladder, stomach and colon, newer intra- and transluminal procedures could be developed. The potential advantages of this approach would include operating within a localized pneumoviscerum environment (e.g., pneumovesicum, pneumogastrum or pneumocolum) in contrast to a generalized pneumoperitoneum, thereby potentially allowing certain major abdominal procedures to be performed under regional rather than general anaesthesia[57]

The priorities for the development to meet future needs of MISS surgery look evenly divided across all four categories – ports, instruments, optics and robotics. Of late, there has been a new entry to MISS surgery platforms: Single-Port Instrument Delivery Extended Research (SPIDER™ developed by TransEnterix Inc., Research Triangle Park, NC, USA). It is proposed that by instrument manipulation past the level of the skin and fascia, the local wound inflammation would be minimized compared with standard laparoscopy. This 18-mm cannula has a retractable outer sheath, four working channels and three distinct ports for insufflation or smoke evacuation. The two lateral flexible channels called instrument delivery tubes provide 360° of freedom at the distal end. Two rigid channels, superiorly and inferiorly, can accommodate an endoscope or any of the rigid surgical instruments with a dimension of <6 mm. Valves maintain pneumoperitoneum. There is a support arm accessory to mount and stabilize the device. In a survival swine model of four cholecystectomies each with SPIDER™ system and conventional four-port laparoscopy, Pryor *et al*. established the feasibility of the SPIDER™ system.[58] Adding further, the SPIDER™ system resulted in minimal tissue trauma compared with the standard four-port laparoscopic cholecystectomy. We need more studies to be sure of its superiority.

### Hybrid NOTES™

**Figure 6:**
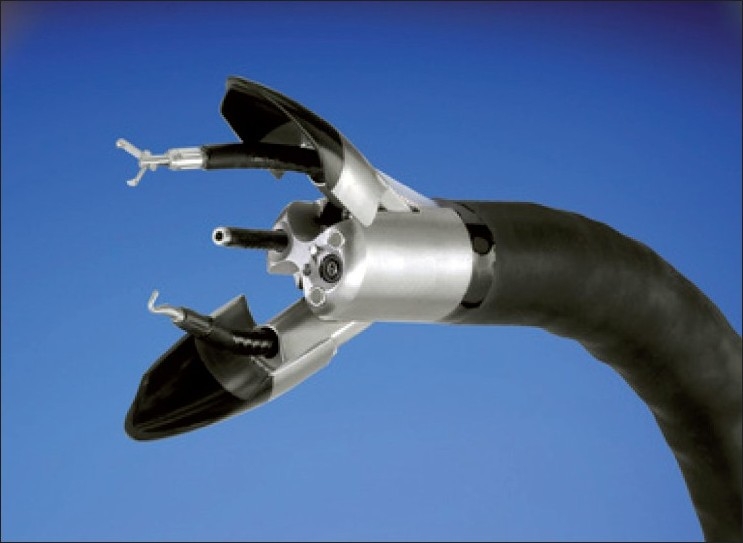
Anubis scope^®^ (Karl Storz, Germany)

Another form of MIS surgery that is rapidly evolving is NOTES™. Surgeons from our institute (IRCAD, Strasbourg) performed the first transvaginal NOTES™ cholecystectomy in humans in 2007.[59] Since then, many NOTES™ procedures have been performed for varied indications using one or two instruments for dissection and retraction introduced through the transumbilical rigid trocars. They are primarily labeled as “Hybrid–NOTES.” It is a win–win situation for both of these surgical access techniques (MISS and NOTES™) as they compensate for the disadvantages of each other and still adhere to the concept of “scarless” surgery. The surgeons at our Institute (IRCAD, Strasbourg) are working towards setting up of protocols for new applications of this concept.[60] A new reusable, multipurpose platform for endoluminal and transluminal procedures called ANUBIS^®^ was developed in collaboration with KARL STORZ and Co. KGTuttlingen, Germany. [Figure 6]. With further development in technology, this hybrid access concept can be established as routine practice. It will be of special value in performing advanced surgical procedures in a minimally invasive manner.

### Robotics for MISS surgery

To develop the field of MISS surgery to the next level, computer-assisted robotic technologies represent a paradigm-changing advance that could potentially resolve the problems imposed by restricted range-of-motion and in-line work and vision.[5] By performing the first transatlantic robot-assisted telesurgery, the authors have unlocked many new doors for evolution of minimally invasive surgery. Robotics forms a mandatory element of telesurgery.[61]

A team of urologists from GUKI, Cleveland showed that robotic single-site surgery is feasible and effective, using the current robotic system; however, with considerable limitations.[57]

**Figure 7:**
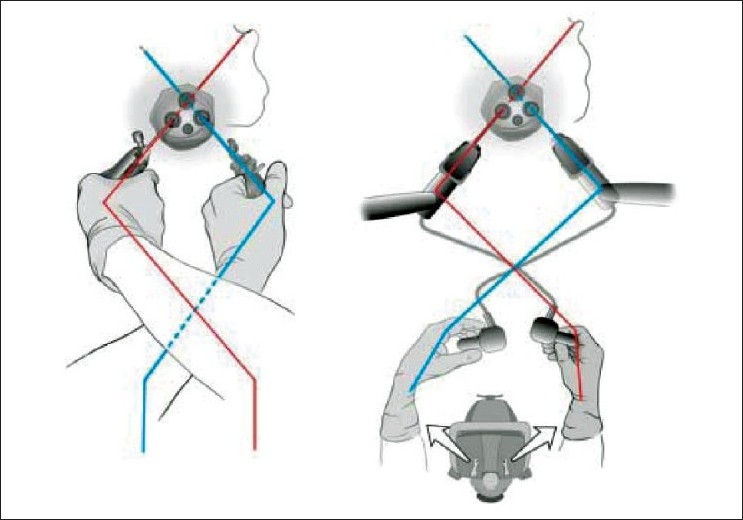
Switch over of robotic arms can rectify for crossing of hands. Very useful for minimally invasive single-site surgery

In the animal study, the surgeons from our institute (IRCAD, Strasbourg) have demonstrated clear benefits of the existing robotic platform (da Vinci^®^ Surgical System; Intuitive Surgical, Sunnyvale, CA, USA) for MISS surgery. Use of the robotic platform allows the surgeon to select which hand will move which instrument. Inverting the control allows crossing of the instruments without any consequence to the surgeon. Moreover, this system offers instruments with multiple degrees of freedom. As a result, robotics may play an essential part in the diffusion of MISS surgery[62] [Figure 7].

### Miniature robots

The miniature robots used for laparoscopic surgery can be divided into three types: imaging robots, lighting robots and retraction robots. The peritoneum-mounted robot or mobile camera robot design are the two options for image feedback.[63] They can be of value in MISS surgery as they reduce the number of items entering the single-site incision.

The feasibility of image feedback from the micro-robot camera for MISS surgery was demonstrated in animal experiments.[64] The pan and tilt micro robot (15 mm diameter × 3 inches length) is capable of rotation along two separate axes, which allows it to pan the operative field through 360° and tilt upward or downward at 45°. The tethers come out by the side of the umbilical port. The second prototype used was the mobile crawler. It consists of two cylindrical wheels capable of navigating to the desired locations in the abdominal cavity. It is equipped with a lens in the middle of the cylinders, providing close-up views of the surgical site. Both the microrobots are controlled by joysticks and were used as a supplement to conventional laparoscopic imagery. One or multiple robots may be used to perform specific tasks or multiple activities during a particular procedure. They may aid not only with visualization but also with manipulation of the surgical field, eliminating the need for multiple port placement. Furthermore, they can potentially be equipped with sensors to provide feedback from the abdomen or surgical site.

MAGS cautery dissectors controllable by a joystick outside have been developed and successfully tested on animal models.[65] Combining various individual applications of miniature robots in minimally invasive surgery, Lehman *et al*. developed a bidextrous robot that is completely inserted into the peritoneal cavity through a single transabdominal incision or a natural orifice, anchored to the abdominal wall and manipulated from the outside for performing surgery.[66] They demonstrated its feasibility for performing single-incision, advanced laparoscopic surgery. The dexterous robot design, 26 mm in diameter, consists of a left grasper and a right cautery forearm, each connected to a central body at a shoulder joint link. Once inserted, the robots provide a stable platform for visualization with sufficient dexterity, triangulation and speed to perform surgical tasks from multiple orientations and workspaces within the peritoneal cavity. Dexterous miniature robots can provide an alternative approach for addressing the limitations of MISS surgery.

### Computer-assisted surgery

With major developments in the fields of imaging, data processing, simulation and virtual reality, we can foresee computer-assisted surgery playing a major role in creating new opportunities for MISS surgery. Our center (IRCAD, Strasbourg, France) has been steering this evolution ahead by presenting some of the pioneering works in this field. A major step in this evolution will be the patient-specific simulation and creation of a “virtual model” of organ-systems that can allow real-time monitoring of the surgical process.[67] Presently, surgical simulation is helping the surgeons in pre- and intraoperative planning and training. But, in the future, this can virtually diminish the surgeon’s dependence on camera vision by providing a reconstructed image of the operative field and can improve dexterity of the instruments. Thus, it will be possible to eliminate the technical limitations we face today in MISS surgery by being able to do what is beyond the human capability.

Thus, we conclude that there are many existing novel technology innovations and many with a huge potential to help MISS surgery mature. Taking into consideration the developments on the NOTES™ front, these two forms of MIS are sure to merge in the near future. We are of the opinion that the “Ultimate form of Minimally Invasive Surgery” will be a hybrid form of MISS surgery and NOTES™ complimented by the technological innovations from the field of robotics and computer-assisted surgery. Thus, the complete spectrum of advanced surgical procedures will be possible with this concept of “scarless surgery.” The future looks exciting and promising. But, we need to move cautiously and wait for the development of appropriate technology and its inculcation into clinical practice.
